# Estimating Health Expectancy in Japanese Communities Using Mortality Rate and Disability Prevalence

**DOI:** 10.31662/jmaj.2023-0058

**Published:** 2023-12-27

**Authors:** Rikuya Hosokawa, Toshiyuki Ojima, Tomoya Myojin, Jun Aida, Katsunori Kondo, Naoki Kondo

**Affiliations:** 1 Department of Human Health Sciences, Graduate School of Medicine, Kyoto University, Kyoto, Japan; 2 Department of Community Health and Preventive Medicine, Hamamatsu University School of Medicine, Shizuoka, Japan; 3 Department of Public Health, Health Management and Policy, Nara Medical University, Nara, Japan; 4 Department of Oral Health Promotion, Graduate School of Medical and Dental Sciences, Tokyo Medical and Dental University, Tokyo, Japan; 5 Center for Preventive Medical Sciences, Chiba University, Chiba, Japan; 6 Center for Well-being and Society, Nihon Fukushi University, Aichi, Japan; 7 Center for Gerontology and Social Science, National Center for Geriatrics and Gerontology, Aichi, Japan; 8 Graduate School of Medicine and School of Public Health, Kyoto University, Kyoto, Japan

**Keywords:** health expectancy, life expectancy, mortality, disability, regression analysis

## Abstract

**Introduction:**

Although mortality and disability are known to be associated with health expectancy (LE), few studies have assessed the extent to which a reduction in their prevalence can extend a person’s LE. Moreover, differences in this relationship based on gender have not been established. Thus, in this study, we constructed a regression model using the rate of mortality and prevalence of disability to predict LE in older adults (≥65 years) and assess the relationships between LE, mortality rate, and disability prevalence based on gender.

**Methods:**

Data were collected from Japan’s population registry and long-term insurance records (N = 344). Multiple linear regression was used to analyze the relationship between LE, mortality rate, and disability prevalence, stratified by gender.

**Results:**

Age-adjusted mortality rate and disability prevalence significantly predicted LE and were significantly correlated with the measured LE index for both genders. For every 1% annual decrease in age-adjusted mortality, LE increased by 1.54 years for men and 2.15 years for women. Similarly, a 1% annual decrease in age-adjusted disability prevalence increased LE by 0.22 years for men and 0.32 years for women. The regression model coefficients indicated that the strength of the association between LE, mortality rate, and disability prevalence differed between genders. Our model accurately predicted LE (men: adjusted R^2^ = 0.968, women: adjusted R^2^ = 0.994).

**Conclusions:**

Health promotion policies that are geared toward increasing health expectancy can be evaluated using mortality rate and disability prevalence as prognostic indicators. The strength of the association between LE, mortality, and disability differed between genders, suggesting the need for gender-specific policy planning to increase LE for both genders.

## Introduction

In most countries, life expectancy (LE) continues to increase steadily and is used as a measure for assessing a population’s health. However, LE is an inadequate measure because it does not capture individuals’ quality of life (QoL) in their later years ^(1)^. Therefore, surveying health expectancy (HE) with an emphasis on the individual’s anticipated QoL has become a common focus over recent years ^(2), (3), (4), (5)^. HE covers the period during which individuals live without any health-related encumbrances in their daily lives (i.e., the average period spent without impediments to their daily activities). Presently, Japan has the highest LE worldwide. According to the World Health Organization, Japan had an estimated average LE of 84.2 years at birth in 2016 ^(1)^. It also has the highest HE worldwide, with an average estimate of 74.8 years in 2016. The difference between LE and HE indicates the average number of years lived in poor health. Thus, on average, Japanese individuals spend the last 9.4 years of their lives with health-related limitations in their daily activities. This gap is significant, suggesting that a long lifespan does not necessarily correspond to a higher QoL.

Many countries other than Japan have major health policy concerns regarding the extent to which QoL improvement is on par with increased LE ^(1)^. Despite health improvements worldwide, more populations are spending longer periods with functional health loss. The prevalence of increased HE is lower than that of LE, resulting in longer periods of poor health and indicating a general increase in morbidity ^(2), (3), (5)^. Consequently, in recent years, countries such as Japan have been implementing various policies to extend HE. The number of years spent in good or poor health has important implications for policies and budgets. In Japan, an HE extension plan is included in the national health policies, representing one of the main goals of the 10-year (2013-2022) nationwide project for health promotion, the Second Term of the National Health Promotion Movement in the 21^st^ Century, established by the Ministry of Health, Labour and Welfare ^(6), (7)^. Reducing the difference between the average LE and HE will prevent a decline in the QoL of individuals and reduce the burden on the social security system.

HE indicators are also of great interest for policy monitoring, as they can be used to objectively measure the effect of policies established to increase health status across regions and for different periods. This enables the assessment of epidemiological patterns and health system performance ^(4)^. Therefore, HE is an important index for evaluating the progress of health promotion plans and strategies enacted by local governments ^(8)^. However, HE is not sufficient as a sole evaluation index for healthcare and welfare policies. An index that can accurately assess goals is required to evaluate policies. It must have a clear causal relationship with the relevant policies and be highly sensitive to them ^(9), (10)^. Even though HE is influenced by various factors, empirical studies related to its measurement capability are scarce ^(2), (11)^. Ideally, indicators should respond quickly and noticeably to changes in the population’s health status. However, HE does not fluctuate significantly during short periods, thereby negatively affecting its sensitivity to the effects of policy changes. Therefore, while HE should be regarded as the main outcome when evaluating policies based on indicators, other related indexes should be used concomitantly with HE.

Several studies have provided evidence of factors that influence the increasing LE trend, such as improvements in living conditions, income, education, medical practices, and regional characteristics ^(12), (13), (14)^. However, strategies to extend HE remain elusive and require further investigation ^(15), (16), (17)^. HE involves an analysis of healthy and unhealthy years, in which health can be defined across various dimensions. Generally, HE combines data on mortality and disability (people who require assistance to perform essential activities of daily life) ^(18)^ to estimate the number of years a population is expected to live in good health or without disabilities. It summarizes mortality and non-fatal outcomes (e.g., chronic health conditions, mobility-related disabilities, and bedridden individuals) as a measure of the average population health. Determining the extent of reduction in mortality rate and disability prevalence is important to extend HE and achieve optimal planning, along with the evaluation of related policies within a given period. Although mortality and disability are associated with HE, few studies have estimated the extent to which a reduction in their prevalence can extend HE. Moreover, it is unclear whether there are gender differences in this relationship. Therefore, this novel study investigates the extent to which a reduction in the mortality rate and prevalence of disability can predict the extension of HE among older adults. Hence, we aimed to construct a regression model using mortality rate and disability prevalence to predict the HE index for men and women separately.

## Materials and Methods

In Japan, administrative units smaller than prefectures were established to provide health services more efficiently (i.e., secondary medical areas), dividing the country into 344 areas as of 2017 (population: minimum-maximum, 20,603-2,691,185 people; mean, 369,461.47 people). They are defined as medical administrative areas under the Medical Care Law and are expected to provide general health services and medical care supplies, such as beds, for inpatients ^(19), (20)^. They play a key role in district-level planning and evaluating HE extension policies. We consequently estimated HE, mortality rate, and functional disability prevalence across these areas. Moreover, the Japanese long-term care insurance system provides care for individuals with functional disabilities for extended periods ^(21)^. We evaluated care needs using the levels certified by this insurance system. Generally, this service is intended for older adults (≥65 years). We calculated HE, mortality rate, and functional disability prevalence for both genders separately.

### Health expectancy

HE can be defined and estimated in various ways. One of these is the Sullivan method, which estimates HE using age-specific death rate and the span of life with a disability prevalence ^(22)^. We used it to calculate HE among older adults in secondary medical areas, stratified by gender. According to this method, HE is determined by the number of person-years lived in good health by applying the age-specific prevalence of an individual’s health status to a life table function (i.e., the number of person-years lived in each age interval). We defined HE as a measure of population health that estimates the expected number of healthy years (i.e., years spent in good health) at a given age.

We then calculated HE using the latest available 2017 population data obtained from Japan’s resident registry ^(23)^. Mortality data were obtained from the vital statistics of total deaths from 2016 to 2018 ^(24)^. Data on care needs were obtained from the report on long-term care insurance services ^(21), (25), (26)^. In this study, “healthy” was defined as the period without impediments to daily activities, whereas “unhealthy” was defined as the period with limitations in daily activities. The Japanese care system is divided into care levels 1-5, based on individual care needs, and is certified by its long-term care insurance system ^(21), (25)^. Care level 1 is for people who experience mild difficulty in performing essential activities of daily life by themselves, whereas care level 2 is for those who require more care to perform these activities. According to this care system, the higher the level, the greater the need for care and the higher the dependence level. Care level 5 is for people who require almost constant care to continue living. Data on unhealthy individuals, including those in care levels 2-5 (with 5 being “almost bedridden”), were obtained from the 2017 long-term care insurance data ^(26)^. This study classified people in level 2 and higher as “having care needs” (i.e., unhealthy). People not classified at any level (i.e., those who did not require any care) and those at care level 1 were classified as having “minimal care needs” (i.e., healthy).

### Mortality rate and disability prevalence

We calculated the annual mortality rate and disability prevalence for older adults, including the crude and age-adjusted mortality rate and the crude and age-adjusted disability prevalence, respectively. The age-adjusted mortality rate is the weighted average of the age-specific death rate in an observed population. The weight for each age category is derived from the proportion of people in the same age category within the general population. We used the general population’s age distribution to adjust the rate and prevalence so that the studied population reflected the same age distribution. Therefore, the rate and prevalence represented summary measurements adjusted for differences in age distributions. We used the 2015 population of Japan as the general population for this study ^(6)^. The crude rate and prevalence are affected by age distribution; therefore, when the latter increases, it may simply reflect aging. The use of age-adjusted prevalence allows comparisons across two or more different periods while removing the effects of changes in age distribution.

### Ethical approval

All data used in this study were exempted from committee approval and informed consent because the Japanese data are freely accessible and available in the public domain.

### Analytic strategy

To analyze the relationship between HE, mortality rate, and disability prevalence, we constructed the following regression model: [HE] = a_1_ × [mortality rate] + a_2_ × [disability prevalence] + a_0_. Associations between HE and the aforementioned variables were assessed using multiple linear regressions, stratified by gender. This was performed to evaluate the extent to which the mortality rate and functional disability prevalence could act as predictors of the HE outcome. Regression analyses were organized as follows: Model 1 was an unadjusted model based on crude mortality rate and functional disability prevalence, while Model 2 was an adjusted model based on mortality rate and functional disability prevalence tailored according to age. The correlation between the predicted (calculated using the regression model) and measured values was determined using the *R*-value. Multicollinearity was assessed using the variance inflation factor. There was no multicollinearity among the predictors (variance inflation factor < 2). We used IBM’s SPSS Statistics for Windows, version 26 (IBM Corp., Armonk, NY, USA) software for all statistical analyses.

## Results

### LE, HE, and unhealthy LE for older adults

Table 1 shows the LE, HE, and unhealthy LE values for older adults. The average HE for men and women aged 65 years and older was 17.70 years (minimum-maximum, 15.78-19.04 years; regional difference, 3.26 years) and 20.83 years (minimum-maximum, 19.37-22.44 years; regional difference, 3.07 years), respectively. The regional difference in HE between secondary medical areas (i.e., between medical areas with the highest and lowest HE) was approximately three years.

**Table 1.** Life Expectancies (in Years) for Older Adults.

Variables (years)	Men				Women			
	*M*	*SD*	*Min*	*Max*	*M*	*SD*	*Min*	*Max*
Life expectancy	19.22	0.52	17.66	20.73	24.08	0.50	22.19	25.28
Health expectancy	17.70	0.53	15.78	19.04	20.83	0.52	19.37	22.44
Unhealthy life expectancy	1.52	0.19	0.95	2.03	3.25	0.34	2.27	4.57

*Note.* Life expectancy, health expectancy, and unhealthy life expectancy were calculated using the Sullivan method. M: mean, SD: standard deviation, Min: minimum value, Max: maximum value

### Mortality rate and disability prevalence for older adults

Table 2 shows the crude mortality rate and disability prevalence, age-adjusted mortality rate, and age-adjusted care-needs certification prevalence. The average age-adjusted mortality rate was 0.047 for men (minimum-maximum, 0.040-0.056; regional difference, 0.016) and 0.028 for women (minimum-maximum, 0.024-0.035; regional difference, 0.010). The regional difference in age-adjusted mortality rate between secondary medical areas (i.e., between medical areas with the highest age-adjusted mortality rate and those with the lowest age-adjusted mortality rate) was approximately 1%. The average age-adjusted long-term care certification prevalence was 0.077 for men (minimum-maximum, 0.046-0.110; regional difference, 0.064) and 0.099 for women (minimum-maximum, 0.064-0.138; regional difference, 0.074). The regional difference in age-adjusted long-term care certification prevalence between secondary medical areas (i.e., between medical areas with the highest and lowest age-adjusted long-term care certification prevalence) was approximately 7%.

**Table 2.** Mortality Rate and Disability Prevalence in Older Adults.

Variables	Men				Women			
	*M*	*SD*	*Min*	*Max*	*M*	*SD*	*Min*	*Max*
Crude mortality rate	0.042	0.004	0.033	0.051	0.033	0.004	0.024	0.043
Age-standardized mortality rate	0.047	0.003	0.040	0.056	0.028	0.002	0.024	0.035
Crude disability prevalence	0.069	0.010	0.044	0.102	0.120	0.018	0.081	0.173
Age-standardized disability prevalence	0.077	0.010	0.046	0.110	0.099	0.011	0.064	0.138

*Note.* M: mean, SD: standard deviation, Min: minimum value, Max: maximum value

### Associations between HE, mortality rate, and disability prevalence

**Figure 1.**
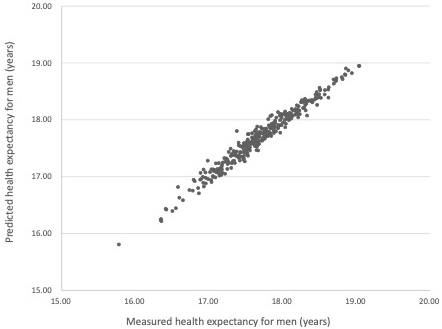
Association Between Measured and Predicted Health Expectancy (HE; Using the Age-Adjusted Model) for Men *Note*: Predicted HE = −154.208 × [age-adjusted mortality rate], −21.536 × [age-adjusted disability prevalence] + 26.682.

Table 3 shows the associations between HE, mortality rate, and functional disability prevalence in men. In Model 1 (crude model), HE at 65 years for men = −44.757 × [crude mortality rate], −15.254 × [crude disability prevalence] + 20.647, and *R* was 0.550. In Model 2 (age-adjusted model), HE at 65 years for men = −154.208 × [age-adjusted mortality rate], −21.536 × [age-adjusted disability prevalence] + 26.682, and *R* was 0.984. The regression analysis using age-adjusted values indicated a high correlation between predicted and measured HE (Figure 1), including high R^2^ and adjusted R^2^ values.

**Table 3.** Association of Health Expectancy with Mortality Rate and Disability Prevalence in Men.

	*B*	*SE*	*β*	*p*	*R*	*R^2^*	*Adjusted* *R^2^*
**Model 1**
Mortality rate	−44.757	7.541	−0.327	<0.001	0.550	0.303	0.299
Disability prevalence	−15.254	2.857	−0.294	<0.001
(Constant)	20.647	0.265	－	<0.001
**Model 2**
Mortality rate	−154.208	1.977	−0.794	<0.001			
Disability prevalence	−21.536	0.556	−0.395	<0.001	0.984	0.968	0.968
(Constant)	26.682	0.091	－	<0.001

*Note.* Model 1 is the crude model. Model 2 is the age-adjusted model. B: unstandardized coefficient, SE: standard error, β: standardized coefficient, R^2^: coefficient of determination

**Figure 2.**
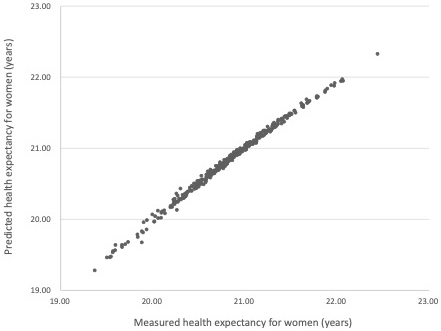
Association Between Measured and Predicted Health Expectancy (HE; Using the Age-Adjusted Model) for Women *Note:* Predicted HE = −215.191 × [age-adjusted mortality rate], −31.900 × [age-adjusted disability prevalence] + 29.936.

Table 4 shows the associations between HE, mortality rate, and functional disability prevalence in women. In Model 1 (crude model), HE at 65 years for women = 49.142 × [crude mortality rate], −14.172 × [crude disability prevalence] + 20.890, and *R* was 0.349. In Model 2 (age-adjusted model), HE at 65 years for women = −215.191 × [age-adjusted mortality rate], −31.900 × [age-adjusted disability prevalence] + 29.936, and *R* was 0.997. Similar to the results for men, the regression analysis using age-adjusted values indicated a high correlation between predicted and measured HE (Figure 2), including high R^2^ and adjusted R^2^ values.

**Table 4.** Association of Health Expectancy with Mortality Rate and Disability Prevalence in Women.

	*B*	*SE*	*β*	*p*	*R*	*R^2^*	*Adjusted* *R^2^*
**Model 1**
Mortality rate	49.142	9.564	0.362	<0.001			
Disability prevalence	−14.172	2.074	−0.482	<0.001	0.349	0.122	0.117
(Constant)	20.890	0.235	－	<0.001			
**Model 2**
Mortality rate	−215.191	1.401	−0.637	<0.001			
Disability prevalence	−31.900	0.204	−0.650	<0.001	0.997	0.994	0.994
(Constant)	29.936	0.040	－	<0.001

*Note.* Model 1 is the crude model. Model 2 is the age-adjusted model. B: unstandardized coefficient, SE: standard error, β: standardized coefficient, R^2^: coefficient of determination

## Discussion

In this study, we constructed a regression model using data on mortality rate and disability prevalence in Japan to determine whether HE could be predicted using these parameters. Our findings showed that HE calculated using age-adjusted mortality rate and disability prevalence was strongly correlated with the measured HE. An annual 1% reduction in age-adjusted mortality rate increased HE by 1.54 and 2.15 years for men and women, respectively. Similarly, an annual 1% reduction in age-adjusted disability prevalence extended HE by 0.22 and 0.32 years for men and women, respectively. Adjusting the rate and prevalence by age facilitated more accurate comparisons between groups with different age distributions, as we used the age distribution of the general population to adjust for the studied parameters, and the studied population reflected the same age distribution. Therefore, the mortality and disability parameters provided summary measurements adjusted for differences in age distributions. Although reducing the mortality rate and prevalence of disability is a daunting challenge, several studies have presented evidence regarding individual factors, regional characteristics, and approaches to factors related to mortality ^(27), (28), (29), (30), (31), (32), (33), (34)^ and functional disability ^(35), (36), (37), (38), (39), (40), (41)^. Our study provides useful information for policymakers seeking to extend HE. Therefore, we believe that, while the concept of healthy LE is easy to understand, the actual target values would be more conducive to extending healthy LE if mortality and disability rates were also used.

We also found that the strength of the association between HE, mortality, and disability differed between men and women. Specifically, for men, the association between mortality and HE (β = −0.794, <0.001) was stronger than that between disability and HE (β = −0.395, <0.001). For women, the association between mortality and HE (β = −0.637, <0.001) was similar to that between disability and HE (β = −0.650, <0.001). In previous studies, the mortality rate for men was higher in all age groups. However, women are more likely to experience disabling conditions, with older women having a higher prevalence of decline in physical functions ^(42), (43), (44), (45), (46), (47)^. For instance, data from Japan and other countries showed that the mortality rate for most cancer types was higher among men ^(48), (49)^. Additionally, a study on diseases causing impediments in daily activities in the Japanese population reported that men had a higher prevalence of cerebrovascular diseases, hypertension, and diabetes, whereas women had a higher prevalence of orthopedic diseases such as osteoporosis, arthropathy, frozen shoulder, backache, and rheumatoid arthritis ^(50)^.

The major causes for certification for long-term care in Japan among men are conditions associated with a high risk of death, such as stroke; conversely, the major causes for women involve conditions with a high risk of decreased QoL and living functions, such as dementia and bone fractures ^(51)^. These findings indicate that while men have shorter longevity, women experience more challenges related to physical functioning. Moreover, men and women tend to require long-term care for different reasons, and physical discomfort is reported more frequently by women ^(44), (45)^. Furthermore, in this study, the age-standardized mortality rate was higher in men, whereas the age-standardized disability prevalence was higher in women. Although the exact underlying physiological mechanism of these differences remains unknown, the literature has consistently reported that the mechanism regarding the types and severity of diseases differs in women and men ^(52)^. Women have longer lifespans than men, which contributes to women’s poorer health. This may be reflected in the strength of the relationship between the studied mortality rate, disability prevalence, and HE. Nonetheless, this relationship is complex, and further detailed investigations of the aforementioned trends, stratified by gender, are required for enhanced understanding.

Our study has several strengths. Our findings, derived from data obtained from a credible Japanese statistical administrative database, suggest that mortality rate and disability prevalence can be used as prognostic indicators regarding HE extension ^(53), (54)^. We constructed a regression model using mortality rate and disability prevalence as surrogate indicators and demonstrated that an age-adjusted regression model accurately predicted HE.

### Limitations

Our study has a few limitations; therefore, our findings should be interpreted cautiously and prudently. “Healthy” status was evaluated based on the presence or absence of certification for long-term care ^(21), (25)^. Compared with other health indicators, long-term care certification does not include mental and social factors and is mainly limited to physical factors. Although the care level classification was based on objective data evaluation, it was derived from a Japan-specific system ^(21), (25)^. Thus, generalization to other countries may be limited. Healthy and unhealthy statuses should also be evaluated using other, more readily generalizable criteria. Moreover, in this study, the mortality rate was calculated using the total number of deaths. Therefore, we did not examine how disease-specific mortality rates (such as those for malignant neoplasms, heart disease, and cerebrovascular diseases) affect HE prediction. Future studies should determine the association between disease-specific mortality rate and HE.

### Conclusions

Our study showed that HE can be predicted using a regression model based on the rate of age-adjusted mortality and the prevalence of long-term care certification. For every 1% annual decrease in age-adjusted mortality, HE increased by 1.54 years for men and 2.15 years for women. Similarly, a 1% annual decrease in age-adjusted disability prevalence increased HE by 0.22 years for men and 0.32 years for women. Adjusting rates and prevalence by age allowed for more accurate comparisons between groups with different age distributions. The study population reflected the same age distribution because the parameters were adjusted using the age distribution of the general population. Therefore, the mortality and disability parameters provided summary measures adjusted for differences in age distribution. Accordingly, we believe that, while the concept of healthy LE is easy to understand, the actual target values would be more conducive to extending healthy LE if mortality and disability rates are also used. Age-adjusted mortality rate and functional disability prevalence can be surrogate indicators when evaluating policies for HE extension. Furthermore, the strength of the association between HE, mortality, and disability differed between men and women, suggesting the need for gender-specific policy planning to increase HE for both genders.

## Article Information

### Conflicts of Interest

None

### Sources of Funding

This work was supported by [the Health Labor Sciences Research Grants] grant numbers [19FA1012, 19FA2001, and 22FA1010].

### Acknowledgement

We are grateful to the Health Labor Sciences Research Council for funding this study.

### Author Contributions

NK was responsible for the acquisition of funds. RH and TO were responsible for the research design and investigation, methodology, resources, software used, validation and visualization of data, and writing the original draft of the manuscript. TO supervised the study. NK was responsible for project administration. TM, JA, KK, and NK were responsible for reviewing and editing the manuscript. All authors have approved the final version of the manuscript.

### ORCiD iD

Rikuya Hosokawa: https://orcid.org/0000-0003-4239-8494

### Approval by Institutional Review Board (IRB)

All the data used in this study were exempt from committee approval and informed consent because they are freely accessible Japanese data, available in the public domain.
